# Saturated fats, dairy foods and cardiovascular health: No longer a curious paradox?

**DOI:** 10.1111/nbu.12585

**Published:** 2022-10-26

**Authors:** David Ian Givens

**Affiliations:** ^1^ Institute for Food, Nutrition and Health University of Reading Reading UK

**Keywords:** cardiovascular diseases, cholesterol, dairy products, fatty acids, milk, saturated fat

## Abstract

Cardiovascular diseases (CVDs) are a major cause of death and morbidity in many parts of the world, and many dietary guidelines limit the intake of saturated fatty acids (SFA) as they are regarded as an important risk factor for CVDs due to their association with increased blood cholesterol. Dairy foods are often a major contributor to dietary intake of SFA, and since many dietary guidelines contain restrictions on SFA intake, this can lead to a moderation of dairy food intake despite meta‐analyses generally showing dairy to have a neutral or negative association with CVDs. Many prospective studies and randomised controlled trials do not support a simple positive association between SFA intake and the risk of atherosclerotic CVD and its components although some early studies had a number of methodological weakness. Studies that included blood cholesterol data do broadly support the positive relationship between SFA and blood low‐density lipoprotein cholesterol (LDL‐C) but without increased CVD risk resulting, despite LDL being a causal factor in atherosclerotic CVD. These data suggest that LDL‐C alone is not a consistently good predictor or cause of CVD risk, perhaps particularly in relation to dairy food consumption although some non‐dairy food studies have also shown LDL‐C reduction was not reflected in reduced CVD risk. This narrative review examines some reasons for these findings. Overall, restrictions on dairy food intake do not seem warranted, although there remains a need to further understand the association of different dairy food types with chronic diseases, perhaps particularly for type 2 diabetes.

## INTRODUCTION

Over the last 50 years, the UK death rate from cardiovascular diseases (CVDs) has declined substantially from 1050 per 100 000 of adults in 1969 to about 190 per 100 000 adults in 2019 (BHF, 2021) with males still having a higher death rate than females. Life expectancy in the United Kingdom has also increased over the last 40 years, albeit at a slower pace in the last decade (Office for National Statistics, 2022a). Despite the reduction in CVD mortality, this remained the greatest cause of death in UK males in 2018, yet in females from 2011, it has been replaced by dementia/Alzheimer's disease (Office for National Statistics, 2022b). CVDs also account for 45% of all deaths in Europe and 37% of all deaths in the EU and cost the EU economy €210 billion per year (European Heart Network, 2017). It therefore remains critical that modifiable risk factors for CVDs such as diet and exercise are used to maximise and maintain the reduction in CVDs. Moreover, it is increasingly apparent that attention to these risk factors should start in childhood and continue throughout adult life (Arteaga & Gillman, 2020).

For a considerable length of time, a high intake of saturated fatty acids (SFA) has been identified as a critical risk factor for CVDs on the basis that it leads to increased blood low‐density lipoprotein cholesterol (LDL‐C) that in turn contributes to the development of atherosclerosis. This apparently simple model was discussed in an earlier contribution to this topic (Givens, 2017) and is becoming increasingly challenged (Astrup et al., 2019, 2021; Krauss & Kris‐Etherton, 2020; Nestel & Mori, 2022). Despite recommendations that dietary guidelines should become food‐based rather than being nutrient‐specific (Astrup et al., 2020), many guidelines still impose a restriction on SFA intake. In the United Kingdom, revisited guidelines confirmed that SFA intake should not exceed 10% of total energy intake (SACN, 2019) which agrees with the US recommendations (US DHHS, 2015), yet it is noteworthy that the American Heart Association and American College of Cardiology *Guidelines to Reduce Cardiovascular Risk* recommended reducing SFA intake to 5%–6% of total energy intake to reduce circulating LDL‐C (Eckel et al., 2014). The evidence available to the Scientific Advisory Committee on Nutrition (SACN) (2019) had considerable limitations in terms of extent, quality and consistency of study outcomes. For total CVDs, it was reported there was adequate evidence from RCTs that either reducing SFA intake or substituting it with polyunsaturated fatty acids (PUFA) had no effect on CVD mortality. There was also insufficient evidence available from RCTs to determine any effect of substituting SFA with monounsaturated fatty acids (MUFA) on CVD mortality and events. Similarly, there was adequate evidence from prospective cohort studies that intake of SFA was not associated with CVD mortality or CVD events. SACN (2019) did however conclude that there was adequate evidence from RCTs that substituting SFA with PUFA reduced the risk of CVD events with no evidence available from prospective cohort studies. Broadly, similar findings were reported for coronary heart disease (CHD), although most of the evidence was more limited or graded moderate than for CVDs. For total stroke, there was adequate evidence from RCTs of no effect between SFA intake and total strokes with a similar conclusion from prospective studies for ischaemic strokes, although there was limited evidence that lower intake of SFA increased the risk of haemorrhagic strokes although this was only based on Japanese Asian populations living in Japan. The recommendation of SACN to retain the guidance that SFA intake should not exceed 10% of total energy intake was influenced by the RCT findings of adequate evidence that substituting SFA with PUFA reduced the risk of CVD events. It is however important to note that SACN (2019) was considering SFA without reference to the foods that supply them, whereas this review is particularly focused on SFA from dairy foods and evidence is discussed that the food supplier of SFA can influence their effect (e.g. de Oliveira Otto et al., 2012).

As shown in Table 1, the recent mean intake of SFA by sex and age groups in the UK *National Diet and Nutrition Survey* (Public Health England, 2020) exceeds the 10% of total energy intake target, and the contribution of dairy foods (including butter) to SFA intake (27%–33%) is the largest of all food groups. Achieving the SFA target of ≤10% of total energy intake may indirectly result in reduced dairy product consumption. Indeed, the UK Eatwell Guide (Public Health England, 2016), a food‐based dietary guidance introduced in 2016, has a dairy‐containing segment of 21% lower than in the earlier Eatwell Plate (Scarborough et al., 2016). In addition, the dairy‐containing segment in the Eatwell Guide now also contains dairy alternative drinks/foods, which may not be as nutrient dense and functional as dairy products (Clegg et al., 2021). As noted above, recommendations to reduce/limit SFA intake without consideration of the food source are being increasingly challenged. This narrative review is, in part, an update on that of Givens (2017).

**TABLE 1:** Mean UK daily intake of saturated fatty acids (SFA) and as a percentage of total energy (TE) by gender and age (from years 2016/2017–2018/2019), Public Health England (2020)

	Males (years of age)				Females (years of age)			
	4–10	11–18	19–64	65+	4–10	11–18	19–64	65+
SFA (g/day)	22.4	25.4	28.0	26.8	19.8	21.7	22.5	22.8
SFA % total energy	13.0	12.7	12.1	12.9	13.1	12.5	12.4	13.7
Contribution of dairy foods to total SFA intake (%)^a^	33	27	27	33				

^a^ Mean of both sexes and includes SFA from butter.

## EFFECTS OF SATURATED FAT CONSUMPTION ON CARDIOVASCULAR DISEASE EVENTS AND MORTALITY

### Evidence from prospective cohort studies

The meta‐analysis of 21 prospective cohort studies by Siri‐Tarino et al. (2010) involving 347 747 subjects indicated that SFA was not associated with increased risk of CHD (Relative risk [RR] 1.07, 95% confidence interval [CI] 0.96–1.19), CVDs (RR 1.00, 95% CI 0.89–1.11) or stroke (RR 0.81, 95% CI 0.62–1.05). The authors concluded that their meta‐analysis showed that there was still insufficient evidence from prospective studies to conclude that dietary SFA is associated with an increased risk of CHD, stroke or overall CVDs. The meta‐analysis of Chowdhury et al. (2014) involved 32 observational studies with fatty acid intakes and 17 observational studies with data for circulating fatty acids. The relative risk for CHD comparing the highest versus lowest SFA intake was neutral (RR 1.03, 95% CI 0.98–1.07). A similar neutrality was observed for MUFA and *n*‐6 PUFA whilst long‐chain *n*‐3 PUFA was associated with a reduced risk (RR 0.87, 95% CI: 0.78–0.97). Following a meta‐analysis of 12 prospective cohorts, de Souza et al. (2015) also raised serious doubts about the universal validity of SFA being associated with increased risk of CVD. They found no association between SFA intake and all‐cause mortality, CVD mortality or ischaemic stroke with what they described as ‘no convincing lack of association’ between SFA and CHD mortality (RR 1.15, 95% CI: 0.97–1.36, *p* = 0.10). Many would interpret the latter results as simply non‐significant.

The *PURE* study (Dehghan et al., 2017) was a prospective cohort study carried out in 18 countries (three high, 11 middle and four low income) from five continents which examined the association of fat and carbohydrate intakes with all‐cause mortality and a range of CVD‐related events. It involved 135 335 subjects aged 35–70 years with a median follow‐up period of 7.4 years. The key findings in relation to SFA intake are summarised in Table 2, which show that increasing intake of SFA was associated with a significantly reduced risk of total mortality, stroke and non‐CVD mortality but with no association with major CVD events, myocardial infarction (MI) or CVD mortality. Increased intake of MUFA and PUFA was also associated with reduced risk of total mortality but carbohydrate intake was associated with increased risk of total mortality but not risk of CVDs or CVD mortality. The relationships of SFA intake and blood lipids in the *PURE* study (Mente et al., 2017) showed that whilst increased SFA intake increased serum total cholesterol (TC) and LDL‐C it also increased high‐density lipoprotein cholesterol (HDL‐C) leading to a reduction in the TC:HDL‐C ratio, both associated with reduced CVD risk in agreement with the observed reduction in all‐cause mortality and stroke (Mente et al., 2017). In addition, serum triacylglycerols (TAG) declined with increasing SFA intake, which was also associated with reduced CVD risk. The key implication of these findings is that the use of either TC or LDL‐C as CVD risk markers would lead to a completely wrong prediction of CVD‐related risk as highlighted by Astrup et al. (2019). There were, however, limitations in the *PURE* study. In the study overall, the mean contribution of SFA to total energy intake was only 8.1% and diet assessment was only done at baseline using a food frequency questionnaire. In addition, the range in socioeconomic status across the 18 countries was wide which may have been a confounding factor.

**TABLE 2:** Association between percentage energy intake from saturated fatty acids (SFA) and clinical outcomes in the PURE study (from Dehghan et al., 2017)

	Hazard ratio (HR, 95% CI^a^) versus Quintile 1					*p* trend
	Quintile 1	Quintile 2	Quintile 3	Quintile 4	Quintile 5	
% energy from SFA^b^	2.8	4.9	7.1	9.5	13.2	
Total mortality	Reference	0.96 (0.88–1.05)	0.92 (0.83–1.02)	0.85 (0.75–0.95)	0.86 (0.76–0.99)	0.0088
Major CVD events	Reference	1.13 (1.02–1.25)	1.06 (0.95–1.18)	1.03 (0.91–1.17)	0.95 (0.83–1.10)	0.49
Myocardial infarction	Reference	1.28 (1.08–1.51)	1.20 (1.00–1.44)	1.16 (0.95–1.41)	1.17 (0.94–1.45)	0.40
Stroke	Reference	1.10 (0.97–1.25)	1.01 (0.87–1.17)	0.93 (0.78–1.11)	0.79 (0.64–0.98)	0.0498
CVD mortality	Reference	1.04 (0.87–1.24)	0.95 (0.78–1.17)	0.99 (0.79–1.23)	0.83 (0.65–1.07)	0.20
Non‐CVD mortality	Reference	0.94 (0.84–1.04)	0.91 (0.81–1.03)	0.78 (0.68–0.91)	0.86 (0.73–1.01)	0.0108

Abbreviation: CVD, cardiovascular disease.

^a^ Confidence interval.

^b^ Median of each quintile.

More recently, Heileson (2019) reported an umbrella study of nine meta‐analyses of prospective studies (a total of 147 studies and ~3.1 million subjects). All but two of the meta‐analyses reported that SFA intake was not associated with CHD whilst two concluded that replacement of SFA by PUFA was associated with a reduced risk of CHD although the overall conclusion by Heileson (2019) was that meta‐analysis of observational studies found no association between SFA intake and CHD.

Mazidi et al. (2020) studied the associations between quartiles (Q) of total fat, SFA, MUFA and PUFA intake on the risks for all‐cause, CHD and stroke‐related mortality in 24 144 subjects in the *National Health and Nutrition Examination Surveys* (*NHANES*) 1999–2010. They added data from their own study leading to a meta‐analysis based on published studies up to November 2018. Based on fully adjusted Cox‐proportional hazard models in the *NHANES* prospective study, there were inverse associations of intake (Q4 vs. Q1; 120 vs. 32 g/day) of total fat (Hazard ratio [HR], 0.90, 95% CI: 0.82–0.99) and PUFA (HR 0.81, 95% CI: 0.78–0.84) with all‐cause mortality whilst SFA intake was associated with increased mortality (HR 1.08, 95% CI: 1.04–1.11). These results are from the fully adjusted model (No. 2) which adjusted for serum non‐HDL‐C and dietary cholesterol although the difference this adjustment made was not reported. The meta‐analysis involving 29 prospective cohorts (~1.2 million subjects) showed an inverse association between intake of total fat (HR 0.89, 95% CI: 0.82–0.97), MUFA (HR 0.94, 95% CI: 0.89–0.99) and PUFA (HR 0.89, 95% CI: 0.84–0.94) with all‐cause mortality although SFA showed no significant association (HR 1.05, 95% CI: 0.99–1.12). No associations were seen for total fat (HR 0.93, 95% CI: 0.80–1.08) and SFA (HR 0.93, 95% CI: 0.80–1.08) with CVD mortality. Total fat had no association with CHD mortality (HR 1.03, 95% CI: 0.99–1.09), whereas SFA had a positive association with CHD mortality (HR1.10, 95% CI: 1.01–1.21). Neither MUFA nor PUFA intakes were associated with CVDs or CHD mortality but both had inverse associations with stroke mortality (MUFA HR 0.80, 95% CI: 0.67–0.96; PUFA HR 0.84, 95% CI: 0.80–0.90). No association was seen for SFA and stroke mortality. As the authors mention, single‐nutrient studies are complicated by the fact that changes in one nutrient may affect another. As a result, it is not known if increased MUFA and PUFA intake replaced SFA or whether all were independent changes, which could influence the interpretation. This study does add to the limited data on the beneficial association of MUFA with CVD‐related mortality and indicates a limited association of SFA with the range of CVD‐related outcomes studied.

Kim et al. (2021) reported a further meta‐analysis of 19 studies (~1 million subjects). Overall, this reported a non‐linear association between SFA and all‐cause and CVD mortality with the risk increasing up to an SFA intake of 11% of energy intake followed by a plateau or slightly reducing risk with higher intakes. A sub‐group analysis that included adjustments for fat intake and serum lipids did not change the associations. Significant inverse associations with all‐cause mortality were seen for MUFA and PUFA intakes. Schwingshackl et al. (2021) carried out a scoping study of 44 systematic reviews of prospective studies mostly on the effect of the highest versus lowest exposure to dietary fat/fat types on health outcomes. Overall, this mainly found no association of total fat, MUFA and/or PUFA and SFA with risk of CVDs and CHD and SFA had no association with all‐cause mortality and a negative association with risk of stroke. This study also included a scoping study of an RCT, which is discussed in the next section.

The evidence, therefore, from prospective studies on the association of SFA with mortality and CVD events is mixed although most studies/meta‐analyses (Chowdhury et al., 2014; de Souza et al., 2015; Dehghan et al., 2018; Heileson, 2019; Mazidi et al., 2020; Schwingshackl et al., 2021; Siri‐Tarino et al., 2010) reported a neutral association. The *PURE* study (Dehghan et al., 2018; Mente et al., 2017) is of particular interest as it also provided details on blood lipids. Increased SFA intake increased serum TC and LDL‐C but also increased HDL‐C leading to a reduction in the TC:HDL‐C ratio, in agreement with the observed reduction in all‐cause mortality and stroke. This study indicates that intake of SFA is not a good predictor of CVD risk and that the use of LDL‐C as a risk factor in isolation may also be misleading.

Whilst prospective studies are usually regarded as providing less valuable evidence than data from RCTs, they do have merits including large subject numbers, long study periods, ability to study intake of foods, not just individual nutrients and hard clinical outcomes. Generally, they cannot prove cause and effect, for which RCTs are needed. Nevertheless, as recently highlighted by DuBroff and de Lorgeril (2021) and Astrup et al. (2021), the evidence that prospective studies provide should be seriously considered when dietary guidelines are set since the simple ‘diet‐heart hypothesis is now of uncertain validity’ (DuBroff & de Lorgeril, 2021).

### Evidence from randomised controlled trials

Two early large RCTs in an ambulatory coronary care clinic and a care home plus psychiatric hospital setting were, respectively, the *Sydney Heart Study* (Ramsden et al., 2013) and the Minnesota Coronary Experiment 1968–1973 (Frantz Jr et al., 1989; updated by Ramsden et al., 2016). The *Sydney Heart Study* was conducted from 1966 to 1973. Having recovered the original data, Ramsden et al. (2013) reanalysed the study using modern statistical techniques. The study involved 458 men aged 30–59 years who had had recent coronary events. The intervention group (*n* = 237) had dietary saturated fats reduced to less than 10% of food energy intake (FEI) and *n*‐6 linoleic acid from safflower oil and safflower oil polyunsaturated margarine increased to about 15% FEI whilst the control group (*n* = 237) was given no specific dietary advice. The median follow‐up period was 39 months. The key results showed the PUFA intervention group to have higher mortality rates than the controls on a relatively SFA‐rich diet (all cause 17.6% vs. 11.8%, CVDs 17.2% vs. 11.0%, CHD 16.3% vs. 10.1%) However, as discussed below this study had many weaknesses.

The Minnesota Coronary Experiment was conducted from 1968 to 1973. After recovering the raw data from the study along with various previously unpublished records and study documents, Ramsden et al. (2016) analysed the data according to the originally prescribed hypotheses. The study was a double‐blind RCT aimed at assessing partial replacement of dietary SFA (control diet) using linoleic acid‐rich plant oil (intervention diet) on primary and secondary prevention of cardiovascular events and mortality. Only a limited number (2355) were included in this 2016 analysis. The SFA in the control diet (SFA 18%, PUFA 3.8% energy intake [EI]) was provided mainly by animal fats, margarines and shortenings whilst the linoleic acid in the intervention diet (SFA 9.2%, PUFA 13.2% EI) came from liquid maize oil and polyunsaturated margarine. The intervention treatment led to a 13.8% reduction in TC compared with only a 1% reduction in the control group, yet there was no mortality benefit from the intervention diet.

Overall, the two RCTs (Ramsden et al., 2013, 2016) appeared to contradict the classical diet‐heart hypothesis such that whilst SFA replacement led to reductions in TC (and presumably LDL‐C which was not reported), this did not translate into reduced mortality. Both studies involved a replacement of SFA with a high amount of *n*‐6 linoleic acid meaning the results do not necessarily hold for replacement with other fatty acid families including MUFA and *n*‐3 fatty acids. Indeed, the meta‐analysis of Ramsden et al. (2013) involving four studies which included *n*‐3 fatty acids showed significantly reduced risk of CVD mortality (HR 0.79, 95% CI: 0.63–0.99). Both studies had a range of weaknesses. There must be considerable doubt about the relevancy of the diets involved to those typical today and similarly of some aspects of the subjects (e.g. in the Sydney study ~70% were smokers at baseline). In addition, the Minnesota study was carried out in psychiatric hospitals and nursing homes, and as the authors concede, the results may not apply to populations without mental illnesses or not living in nursing homes. In addition, since both RCTs (Ramsden et al., 2013, 2016) involved the use of margarines and shortenings, which at that time were, as the authors concede, rich sources of industrial *trans* fatty acids (TFA), it must be considered that TFA influenced the outcome of both studies although the concentrations of TFA in the diets were not measured. There may also be speculation that TFA were present in the linoleic acid‐rich intervention oil, and its oxidation status was not reported, and both could have had a role in increased mortality. Overall, there is considerable doubt that these two studies provide much valuable evidence on the diet‐heart hypothesis but they did create a focus on the subject, which will have stimulated more recent studies.

The narrative umbrella review of Heileson (2019) examined the RCT evidence from 10 meta‐analyses published from 2010 to 2017. None of the analyses reported a significant increase in CHD mortality or total mortality linked to SFA intake, but three found decreases in CHD/CVD events related to replacing SFA with PUFA. Heileson (2019) also reviewed the strengths and weaknesses of this set of analyses and identified high between‐study heterogeneity and highlighted that some key confounding variables such as changes in TFA and *n*‐3 fatty acids and potential bias from lack of blinding made collective interpretation of the results difficult. His overall conclusion was that SFA are not independently linked to heart disease, and despite leading to reductions in LDL‐C, replacing SFA with PUFA may not be beneficial. Heileson (2019) also criticised the conclusions to replace SFA with PUFA of the Presidential Advisory paper from the American Heart Association (Sacks et al., 2017) as the studies they used were ‘plagued by design flaws, lacked dietary control of variables other than fat and did not account for TFA restriction in high PUFA groups’.

Recently, Hooper et al. (2020) published an updated Cochrane review and meta‐analysis examining the effects of replacing SFA EI with carbohydrate, PUFA, MUFA and/or protein on mortality and CVD‐related morbidity using data from 15 RCT (involving 16 comparisons) that met strict inclusion criteria and totalled almost 60 000 subjects. The *Sydney Heart Study* was included but the Minnesota Coronary Experiment was excluded due to its short mean follow‐up period. The key results from this meta‐analysis are summarised in Table 3 and show that reducing SFA had little or no effect on all‐cause mortality, CVD‐related mortality, non‐fatal MI and CHD mortality. However, the results on total (fatal or non‐fatal) MI, stroke and CHD events (fatal or non‐fatal) were reported to be unclear as the available evidence was of very low quality. Nevertheless, the meta‐analysis did find that reducing dietary SFA for at least 2 years led to a significant 21% reduction in combined CVD events although this was not linked to the risk of CVD‐related mortality. It is worth noting that only three studies were included in the ‘at least two years’ subgroup, and there was no significant effect of SFA reduction on CVD events in the ‘up to 24 months’, and ‘longer than 48 months’ subgroups. Overall, no significant differences were found between the replacement of SFA energy with that from PUFA or carbohydrates whilst data on energy replacement by MUFA and protein were extremely limited.

**TABLE 3:** Effect of reducing saturated fat intake compared to habitual saturated fat on CVD risk in adults: a summary of key findings from RCT‐based meta‐analysis of Hooper et al. (2020)

Outcomes	Mean follow‐up period (months)	Number of subjects (thousands)	Risk ratio^a^ (95% CI)	Evidence quality (GRADE^b^)	Interpretation
All‐cause mortality	56	56	0.96 (0.90–1.03)	Moderate	Reducing SFA intake probably makes little/no difference to all‐cause mortality
CVD mortality	53	53	0.94 (0.78–1.13)	Moderate	Reducing SFA intake probably makes little/no difference to cardiovascular mortality
Combined CVD events	52	53	0.79 (0.66–0.93)	Moderate	Reducing SFA intake probably reduces the risk of CVDs but with little effect on mortality risk^c^
Total MI	55	53	0.90 (0.80–1.01)	Very low	Reducing SFA intake on risk of MI is unclear because of very low evidence quality
Non‐fatal MI	55	53	0.97 (0.87–1.07)	Low	Reducing SFA intake may have little or no effect on risk of non‐fatal MI
Stroke	59	51	0.92 (0.68–1.25)	Very low	Effect of reducing SFA intake on risk of stroke is unclear because of very low evidence quality
CHD mortality	65	53	0.97 (0.82–1.16)	Low	Reducing SFA intake may have little or no effect on CHD mortality
CHD events	59	53	0.83 (0.68–1.01)	Very low	Effect of reducing SFA intake on risk of CHD events is unclear because evidence quality is very low

Abbreviations: CHD, coronary heart disease; CI, Confidence interval; CVD, cardiovascular disease; MI, myocardial infarction; RCT, randomised controlled trial; SFA, saturated fatty acids.

^a^ Risk in reduced SFA group cf. usual SFA group.

^b^ GRADE Working group.

^c^ Based on plain language key result.

The meta‐analysis of Hooper et al. (2020) also examined the effect of SFA reduction on changes in serum cholesterol. For the studies with suitable data, TC and LDL‐C had mean changes of −0.24 (95% CI: −0.36, −0.13) mmol/L and − 0.19 (95% CI: −0.33, 0.05) mmol/L, respectively, with the greatest reductions seen with replacement by PUFA. Only one study with replacement by MUFA was available for TC change and none for LDL‐C change, which is perhaps surprising. There were no or very limited changes in HDL‐C and lipoprotein (a). The observed changes in TC were not related to the primary outcomes of all‐cause mortality and CVD mortality. TC reduction was only related to CVD events when the reduction was at least 0.2 mmol/L, but this may be important as discussed below. For secondary outcomes of stroke and non‐fatal MI, changes in TC had no significant relationship, but like CVD events, TC reductions of at least 0.2 mmol/L were associated with a lower risk of all MI (fatal and non‐fatal) events, but it must be remembered that the evidence quality for MI outcomes was GRADE low and very low.

Schwingshackl et al. (2021) carried out a scoping study of 15 RCT showing that isoenergetic replacement of carbohydrate by SFA increased blood TC, LDL‐C and HDL‐C but reduced TAG. Replacement of carbohydrate by MUFA and PUFA reduced TC, LDL‐C and TAG but increased HDL‐C. The authors concluded overall that whilst prospective studies (see previous section) found little or no associations of SFA, MUFA or PUFA with CVDs or all‐cause mortality, the evidence from RCT showed that replacement of SFA with MUFA and/or PUFA ‘improves blood lipids and glycaemic control….and current recommendations to replace SFA with MUFA/PUFA seems reasonable’. As discussed above and below, reductions in blood lipids, notably LDL‐C, do not always lead to the reduced CVD risk often assumed.

Overall, the findings from RCTs are broadly in agreement with the conclusions from the prospective cohort studies that the traditional diet‐heart hypothesis may not always be valid, in this author's view.

### Are all saturated fatty acids equal?

Dawczynski et al. (2015) highlighted some of the weaknesses associated with simply looking at the effects of total SFA without considering the possible effects of individual SFA. They examined the meta‐analysis of Chowdhury et al. (2014) and identified that two of the eight included studies focused on CVD risk of C15:0 and C17:0 fatty acids in serum phospholipids which are primarily a marker for intake of milk fat and fat from other ruminant‐derived foods. Dairy products in general are known to have neutral or negative associations with CVD risk and Dawczynski et al. (2015) proposed that these two studies should have been excluded from the meta‐analysis and reanalysed the data of Chowdhury et al. (2014) excluding the two studies (Hallmans et al., 2003; Warensjö et al., 2010) that used C15:0 and C17:0 fatty acids. This produced a positive association between total SFA concentration in blood and coronary events (RR 1.21, 95% CI: 1.04–1.40) highlighting the importance of not including C15:0 and C17:0 along with other SFA. This was supported by findings in The Netherlands *EPIC* cohort (Praagman et al., 2016). This population had a relatively high SFA intake from dairy foods. A lower risk of IHD was associated with higher SFA intake which was mainly related to the sum of 4:0 through to 10:0, 14:0, the sum of 15:0 and 17:0 SFA and other SFA from dairy food sources including butter, cheese, and milk and milk products. These findings suggest that different SFA have different effects and that a good knowledge of the impact that specific foods, such as dairy, can have on CVD risk is important.

Murru et al. (2022) highlighted the risks of assuming that blood and tissue palmitic acid (16:0) concentrations are a reflection of its dietary intake since some palmitic acid is synthesised de novo, primarily from carbohydrate. In healthy subjects, synthesis occurs primality in adipose tissue although certain conditions including insulin resistance and a high dietary intake of carbohydrates, particularly sugars, enhance hepatic de novo synthesis substantially, contributing to hyperlipidaemia (Schwarz et al., 2003). This is supported by studies showing that replacing dietary SFA with refined carbohydrates is associated with increased risk of CVDs (Siri‐Tarino et al., 2015). Murru et al. (2022) concluded that blaming a single nutrient like palmitic acid, which has recognised physiological properties, and suggesting that reducing its dietary intake will reduce CVDs is too simplistic. They also concluded that population guidelines should not focus on single nutrients in isolation but should be based on consideration of the whole diet.

### The saturated fatty acid‐cholesterol‐CVD risk relationship

For a considerable time, LDL‐C concentration in serum/plasma has been measured to predict the risk of CVDs. There is good evidence that most SFA increase LDL‐C and HDL‐C (Mensink et al., 2003), and there is now no doubt that LDL particles have a direct causative role in the development of atherosclerotic CVDs (Ference et al., 2017), and the consensus paper of Borén et al. (2020) explores the complexity of the mechanisms involved. There is, however, increasing uncertainty that lowering LDL‐C concentration by reducing/replacing dietary SFA intake will inevitably lead to CVD risk reduction (Dehghan et al., 2018; Mente et al., 2017). As noted earlier, some of the studies that have contributed to the evidence are flawed for various reasons including being underpowered and poorly designed but there are a number of other possible reasons that contribute to the uncertainty.

Firstly, the ratio of TC:HDL‐C has been shown to provide a better estimate of CVD risk than LDL‐C alone (Quispe et al., 2020) due, at least in part, to its correlation with LDL particle number (Mathews et al., 2012). For this reason and as recommended by Quispe et al. (2020), TC:HDL‐C should be used to provide additional clinical information on LDL‐C in primary prevention particularly in high‐risk diabetic patients. In addition, Griffin et al. (2021) highlighted that there can be considerable variation in serum LDL‐C response to SFA between individuals which may contribute to the uncertainty of the SFA‐CVD risk relationship. Griffin et al. (2021) identified a number of metabolic and genetic reasons for this variation and proposed that a serum biomarker of serum LDL‐C responsiveness to SFA replacement would be of major benefit to the assessment of CVD risk. This again shows an uncertainty in the reliance on LDL‐C as a useful CVD risk predictor.

A further weakness of using LDL‐C as a predictor of CVD risk relates to the fact that LDL‐C concentration comprised the cholesterol present in all LDL particles, yet the small dense LDL particles (sdLDL), whilst containing less cholesterol per particle, are more strongly associated with increased atherosclerosis than the larger less dense LDL particles (Hoogeveen et al., 2014). The RCT of Bergeron et al. (2019) compared diets with high SFA content (~13%–14% EI) with low SFA diets (~7%–8% EI), in which SFA had been partially replaced with MUFA, with the differences in SFA content between the high and low SFA treatments being achieved mainly through the use of high‐fat dairy foods and butter. There was no significant difference in HDL‐C between the two treatments but whilst the high SFA diet increased plasma LDL‐C, this was due entirely to a significant rise in large LDL particles (~577 vs. 542 nmol/L) with no differences in medium, small or very small LDL particles. This suggests that the effect of SFA from red meat, white meat and dairy (which were used in the diets) as would be predicted from their effect on LDL‐C, could be moderated by the lack of effect on the more atherogenic medium and especially small LDL particles. This is potentially a very important finding although further studies of this type are needed to allow translation of robust evidence into public health dietary guidelines, the principles of which are discussed in a position paper from the Academy of Nutrition Sciences (Williams et al., 2021). For example, the RCT of Vasilopoulou et al. (2020), involving subjects consuming SFA‐reduced, MUFA‐enriched (modified) dairy foods, compared with conventional counterparts, found that whilst the modified foods led to a significantly reduced serum LDL‐C response, there were no significant differences in the distribution of small, medium and large LDL particles between dietary treatments.

A cross‐sectional study of 291 healthy Swedish men indicated that dietary fatty acids typically found in dairy foods were associated with less sdLDL particles (Sjogren et al., 2004). In particular, SFA 4:0 to 10:0 and 14:0 (*p* < 0.05) in the diet and 15:0 and 17:0 (markers of dairy fat intake) in serum phospholipids (*p* < 0.05) were associated with fewer sdLDL particles. This study suggests that LDL particle size distribution may be favourably modified by dairy products in the diet or that other dietary SFA have a detrimental effect and milk SFA attenuate this effect rather than have a positive effect themselves, but this needs further investigation.

There is also now increasing evidence that the effect of SFA depends on the food source. For example, the prospective studies of de Oliveira Otto et al. (2012) and Vissers et al. (2019) both showed that whilst a positive association with CVD risk was seen with SFA from meat, this was not the case with SFA from dairy which contains a higher proportion of short and medium chain SFA (4:0 to 12:0) and 14:0 than meat fat. This effect may be related to food‐specific fatty acids or other aspects of the foods, but clearly detailed RCTs are needed to resolve the reasons for this differential effect.

The use of the ratio of TC:HDL‐C to provide an improved estimate of CVD risk is based on evidence that low HDL‐C concentration is associated with increased CVD risk (Sharrett et al., 2001), although drug treatments to raise HDL‐C have not been associated with reduced all‐cause mortality, CHD mortality, MI or stroke (Keene et al., 2014). However, SFA can increase HDL‐C notably when replacing carbohydrates (Brassard et al., 2017), and SFA from butter, but not cheese, have also been shown to increase the cholesterol efflux capacity of HDL in men but not women, although interestingly, the same study showed that MUFA increased the cholesterol efflux capacity of HDL in women but not men (Brassard et al., 2017). This led Griffin and Lovegrove (2018) to question if the increased functionality of HDL is a compensation for the increased LDL‐C from butter consumption. This study of Brassard et al. (2017) is confirmation that the effect of SFA on the LDL‐C/HDL‐C mediated risk of CVD depends on the food source and possibly the sex of the consumer.

In summary, despite LDL particles having a causal role for atherosclerotic CVDs (Borén et al., 2020; Ference et al., 2017), there are clearly a number of reasons why changes in LDL‐C alone may not reliably predict future atherosclerotic CVD risk. Some of this uncertainty will relate to evidence from studies with flawed designs etc., but there are issues that need to be considered. Whilst the ratio of TC:HDL‐C may improve risk prediction, the fact that the functionality of HDL is now known to vary, as does the LDL sub‐fraction distribution adds further uncertainty. Additional validated risk markers are therefore needed with assessment of vascular endothelial function (Vasilopoulou et al., 2020) and broader vascular function (Price et al., 2022) being potentially valuable non‐blood lipid targets.

## DAIRY CONSUMPTION AND CARDIOVASCULAR DISEASES

### Evidence from prospective cohort studies

There have been a good number of meta‐analyses of the outcomes of prospective cohort studies on dairy food consumption and CVDs over the last 20 years. Early meta‐analyses reported that, overall, high milk consumption does not increase the risk RR for CHD compared with low consumers (Elwood et al., 2008, 2010). A further meta‐analysis (Mente et al., 2009) combined prospective cohort and clinical studies and also indicated no significant increase in the RR of CHD in high versus low milk consumers (RR 0.94; 95% CI 0.75–1.13). More recent meta‐analyses, including the more valuable dose–response meta‐analyses, on consumption of dairy foods and primarily the risk of CVDs and type 2 diabetes include those of Guo et al. (2017), Soedamah‐Muthu and de Goede (2018) and Soedamah‐Muthu and Guo (2020). Poppitt (2020) and Nestel and Mori (2022) have published extensive reviews on the subject of dairy food consumption and human health.

Table 4 summarises the findings of dose–response meta‐analyses as reviewed by Givens (2021). Overall, they broadly agree with earlier meta‐analyses that dairy product consumption has a neutral effect on CVD risk although the results do point to an association with reduced risk of stroke and type 2 diabetes with consumption of milk and yogurt, respectively, although it is noteworthy that the recent study of Olsson et al. (2022) in ~80 000 Swedish subjects found that whilst milk consumption was not clearly associated with total stroke, it was weakly and non‐linearly associated with ischaemic stroke and directly associated with haemorrhagic stroke. No associations were seen for fermented milk. These findings highlight the importance of not assuming that all dairy products are the same and the importance of meta‐analyses to examine the evidence from groups of studies. There are few studies examining the association of butter consumption with CVDs and type 2 diabetes, but the dose–response meta‐analysis of Pimpin et al. (2016) indicates no significant association between butter consumption and all‐cause mortality, CVDs, CHD or stroke, although interestingly, there was a significant negative association with type 2 diabetes. This meta‐analysis involved data from few studies (CVDs *n* = 4; CHD *n* = 3; stroke *n* = 3) although 11 cohorts were included with type 2 diabetes as an outcome. Clearly further studies on butter are needed including a better understanding of the SFA‐mediated increased HDL functionality discussed above.

**TABLE 4:** A selection of dose–response meta‐analyses examining the relative risk (RR) of cardiometabolic diseases in relation to consumption of dairy foods

Dairy food	Outcome	RR (95% CI)	References
Milk (per 244 g/day)	All‐cause mortality	1.00 (0.93–1.07)	Guo et al. (2017)
Milk (per 244 g/day)	CVD	1.01 (0.93–1.10)	Guo et al. (2017)
Cheese (per 10 g/day)	CVDs	0.98 (0.95–1.00)	Guo et al. (2017)
Yogurt (per 50 g/day)	CVDs	1.03 (0.97–1.09)	Guo et al. (2017)
Milk (per 200 g/day)	Stroke	0.93 (0.88–0.98)	de Goede et al. (2016)
Milk (per 200 g/day)	Stroke	0.92 (0.88–0.97)	Soedamah‐Muthu and de Goede (2018)
Cheese (per 40 g/day)	Stroke	0.97 (0.94–1.01)	de Goede et al. (2016)
Yogurt (per 80 g/day)	Type 2 diabetes	0.86 (0.83–0.90)	Gijsbers et al. (2016)
Yogurt (per 100 g/day)	Type 2 diabetes	0.94 (0.91–0.97)	Soedamah‐Muthu and de Goede (2018)
Butter (per 14 g/day)	All‐cause mortality	1.01 (1.00–1.03)	Pimpin et al. (2016)
Butter (per 14 g/day)	CVDs	1.00 (0.98–1.02)	Pimpin et al. (2016)
Butter (per 14 g/day)	CHD	0.99 (0.96–1.03)	Pimpin et al. (2016)
Butter (per 14 g/day)	Stroke	1.01 (0.93–0.99)	Pimpin et al. (2016)
Butter (per 14 g/day)	Type 2 diabetes	0.96 (0.93–0.99)	Pimpin et al. (2016)

Abbreviations: CHD, coronary heart disease; CI, confidence interval; CVDs, cardiovascular disease.

An issue not fully resolved concerns the impact, if any, of the fat content of milk and indeed other dairy foods. For example in the meta‐analyses of Guo et al. (2017) and de Goede et al. (2016), only 1/12 and 4/18 studies, respectively, confirmed what the fat content of the milk was. Some studies have compared the association with CVDs etc for high‐ versus low‐fat dairy foods, but there is not consistency as to which foods are high and low fat. Two recent meta‐analyses have tried to add clarity on this, at least for milk. Naghshi et al. (2022) found that high‐fat milk (presumably not fat reduced) was associated with a significantly higher risk of all‐cause and CVD mortality than low‐fat milk (presumably fat reduced) but this analysis was based on only eight studies out of a total of 27. Similarly, Jakobsen et al. (2021) reported that high‐fat milk was positively associated with increased risk of CHD (RR per 200 g 1.08, 95% CI: 1.00–1.16, *p* = 0.04) whilst low‐fat milk was neutral (RR per 200 g 0.96, 95% CI: 0.96–1.13) but again these outcomes were based on only four and three studies, respectively, with evidence graded as low (low‐fat milk) and moderate (high‐fat milk). The importance of fat content remains uncertain.

The *PURE* study (Dehghan et al., 2018) also examined the association between intake of dairy foods (total and milk, yogurt and cheese) with mortality and CVDs across its 136 384 subjects aged 35–70 years from 21 countries across five continents. Higher intake of total dairy (>2 servings/day vs. no intake) was associated with a lower risk of non‐CVD mortality (HR 0.86, 95% CI: 0.72–1.02, *p* **trend** = 0.046), CVD mortality (HR 0.77, 95% CI: 0.58–1.01, *p* **trend** = 0.029), major CVD events (HR 0.78, 95% CI: 0.67–0.90; *p* **trend** = 0.0001), and stroke (HR 0.66, 95% CI: 0.53–0.82, *p* **trend** = 0·0003). Higher intakes of milk and yogurt, but not cheese, were associated with a lower risk of the combination of mortality or major CVD events. Among participants who only consumed whole‐fat dairy foods, a higher intake of total dairy was associated with reduced risk of all‐cause mortality and of major CVDs although a similar inverse association was seen for those that consumed both whole‐fat and low‐fat dairy foods. However, the large variety of subject populations would represent a wide range of socio‐economic status, which may have confounded the findings. In addition, dietary information was only obtained at baseline, and in many countries, this could have changed substantially over the median 7.4 years of follow‐up. Also coupled with the wide variation in dairy food intake between countries, there was likely to be variation in the degree of lactose intolerance, which could have led to a risk of residual confounding.

Whilst the outcomes are broadly in agreement with earlier studies and meta‐analyses, this is believed to be the first study to involve such large and diverse sets of subjects with substantial variation in habitual intake of dairy and other foods between countries but it does have limitations.

Overall, the findings from prospective cohort studies provide no consistent evidence of an increased risk of CVDs with increased consumption of dairy foods despite most of these foods often being a primary dietary source of SFA intake. To many, this is not only counterintuitive but also contrary to the believed well‐established link between SFA intake, serum LDL‐C and CVDs. It was noted earlier that SFA‐induced increases in serum LDL‐C concentration may not always lead to increased CVD risk but there is now emerging evidence that certain characteristics of dairy foods, including the food matrix, can also go some way to explaining this.

### Effects on blood pressure and haemodynamics

In the UK, up to 30% of adults are hypertensive (Townsend et al., 2015), and hypertension is the third biggest risk factor for premature death after smoking and poor diet but is the largest single known risk factor for CVD development, and stroke in particular (Public Health England, 2017). Gene polymorphisms, nutrition, the environment and interactions between these factors contribute to the development of hypertension as does advancing age and associated increased stiffness of the large arteries. Milk and milk‐derived products provide essential micronutrients (e.g. calcium, magnesium, iodine, several B vitamins) and proteins. The main milk protein types, whey protein and casein, have been associated with beneficial hypotensive effects (Kris‐Etherton et al., 2009). An 8‐week RCT (Fekete et al., 2016) showed that whey protein isolate (2 × 28 g/day, mixed with water) had a greater hypotensive effect on 24 h ambulatory systolic (2.0 ± 0.7 mm Hg) and diastolic (2.9 ± 1.1 mm Hg) blood pressures than casein, and the effects were seen on both central and peripheral systolic blood pressures. The hypotensive effect of whey protein has also been observed in an acute postprandial study (Fekete et al., 2018). A number of mechanisms by which milk and its components could lower blood pressure (BP) have been proposed (Fekete et al., 2013). Peptides released during digestion of casein and whey proteins have been shown to have hypotensive effects by inhibiting the action of the angiotensin‐I‐converting enzyme (a key enzyme in the regulation of BP), resulting in vasodilation (FitzGerald & Meisel, 2000), by modulating the release of endothelin‐1 by endothelial cells (Maes et al., 2004) and acting as opioid receptor ligands increasing nitric oxide production which mediates arterial tone (Kris‐Etherton et al., 2009). There is little firm evidence for differential effects of low‐ versus high‐fat dairy foods on hypertension. For example, whilst Engberink et al. (2009) reported an inverse association between low‐fat dairy intake and risk of hypertension in older adults, others have shown that both low‐ and high‐fat milk products have hypotensive effects (Ralston et al., 2012). In addition, results from the *Caerphilly Prospective Study* showed that when compared with non‐milk consumers, men who consumed >586 ml/day of whole milk had on average a 10.4 mm Hg lower systolic BP after a 22.8‐year follow‐up (Livingstone et al., 2013). The recent review of Price et al. (2022) highlighted some inconsistencies between studies such that some show little effect of whey protein on BP (e.g. Kjølbæk et al., 2017) although it seems that factors including baseline BP, weight loss and obesity during the study period may influence the effect on BP (Price et al., 2022).

There is now good evidence that arterial stiffness, especially of the large vessels, is an important predictor of future CVD events (Cockcroft & Wilkinson, 2000) and this can be affected by diet (Kesse‐Guyot et al., 2010). The measurement of carotid to femoral pulse wave velocity (PWV) is regarded as the gold standard for assessing arterial stiffness and can independently predict CVD events (Van Bortel et al., 2012; Vlachopoulos et al., 2010). Livingstone et al. (2013), using data from the *Caerphilly Prospective Study*, showed for the first time in a longitudinal study that dairy product consumption (not including butter) is not detrimental to PWV and related to increased arterial stiffness. Indeed, the measurement of augmentation index, another valuable indicator of arterial stiffness, was 1.9% units lower (*p* = 0.021) in men with the highest dairy consumption (Livingstone et al., 2013). A cross‐sectional study also reported that consumption of dairy foods was negatively associated with PWV (Crichton et al., 2012). The findings of Ribeiro et al. (2018), from another cross‐sectional study, showed that the intake of total dairy foods was inversely associated with carotid to femoral PWV (−0.13 m/s) and pulse pressure (−1.3 mm Hg) with similar outcomes from consumption of low‐fat dairy, fermented dairy and cheese. The systematic review and meta‐analysis of seven cross‐sectional studies (16 433 patients) by Diez‐Fernández et al. (2019) reported that total dairy foods (Effect size −0.03, 95% CI: −0.04, −0.01), and cheese (Effect size −0.04, 95% CI: −0.07, −0.01) were weakly but significantly associated with lower PWV and hence lower arterial stiffness values. Milk consumption was not significantly associated with PWV (Effect size 0.03, 95% CI: −0.01, 0.05).

The effects of dairy foods on BP, arterial stiffness and other aspects of haemodynamics are important in the overall understanding of the impact of dairy consumption and its association with CVD risk.

### Food matrix effects of dairy products on blood lipids

Historically, the nutritional evaluation of foods and diets and their relationship with the health of the consumer has generally been based on separate assessments of individual nutrients (or clusters of nutrients such as total SFA) including protein, fat, carbohydrates and micronutrients. This approach may explain some of the discrepancies between the predicted health effect of a food, based on its nutrient content and its actual health effect when consumed as a whole food. The increasing evidence that the so‐called food matrix needs to be understood to further evaluate the health effect of some dairy foods needs to be acknowledged and taken into account. This topic was extensively reviewed by a working party set up by the Universities of Copenhagen and Reading and the conclusions reported by Thorning et al. (2017). This was also extensively discussed in the earlier paper (Givens, 2017), and mainly updates are discussed here. Various aspects of understanding dairy matrix effects have been updated in more recent publications (e.g. Aguilera, 2019; Feeney et al., 2021; Feeney & McKinley, 2020; Mulet‐Cabero & Brodkorb, 2021; Weaver, 2021).

To date, probably the strongest evidence for a dairy food matrix effect is the well‐documented differential impact of hard cheese and butter. Despite providing the same mass of SFA, these foods led to different blood lipid responses (e.g. Hjerpsted et al., 2011; Vissers et al., 2019). Essentially, butter gave a predictable rise in serum LDL‐C, whereas the cheese led to little, no or negative LDL‐C responses as discussed earlier (Givens, 2017).

Various mechanisms have been suggested as contributing to the apparent beneficial effects of the cheese matrix but the synthesis of calcium‐fatty acid soaps in the small intestine leading to increased faecal fat excretion is a key component as shown by Lorenzen and Astrup (2011), although the presence of calcium soaps in the faeces was not chemically confirmed. In fact, the creation of calcium soaps in the digestive tract resulting from milk consumption has been known for a long time (Bosworth et al., 1918) and more recently confirmed in the in vitro studies of Lamothe et al. (2017). Interestingly, this study also showed that increasing amount of soaps was produced as the calcium to lipid ratio increased and that, at equal calcium to lipid ratios, dairy foods with fairly solid structure (cheese) yielded more soaps than milk or yogurt with a more fluid structure. Furthermore, there was evidence that cheese made from homogenised milk yielded more soaps than that made from non‐homogenised milk, probably related to greater free fatty acid release from the homogenised milk product (Lamothe et al., 2017). This study, albeit using an in vitro digestion model, does add to the understanding of the calcium‐fatty acid saponification process and how different dairy products may influence this.

The fairly new work of Brassard et al. (2017), showing the influence of the dairy matrix on the differential effect of SFA from butter and cheese on the cholesterol efflux capacity of HDL, was discussed above. Clearly, the dairy matrix in its various forms needs a more comprehensive understanding including further data on specific dairy foods, their fat content, methods of processing (e.g. pasteurising vs. UHT treatment of milk), the mechanisms involved and crucially, whether they have a significant chronic effect (positive or negative) on the health of the consumer.

### Partial replacement of SFA in milk fat by modifying the diet of the dairy cow: does it provide health benefits?

Given the long‐standing evidence that replacing SFA with PUFA can reduce serum LDL‐C and CHD events (Mozaffarian et al., 2010; Siri‐Tarino et al., 2015), it would seem intuitive that changing the fatty acid composition of dairy foods to replace some SFA with PUFA could be a valuable strategy to reduce SFA intake whilst retaining the nutritional and potential health benefits of milk. However, the concentration of PUFA in milk fat is very low and is not amenable to being increased by alteration of diet of the dairy cow (Kliem et al., 2019). However, milk fat contains substantial amounts of MUFA (20–23 g/100 g total fatty acids) which is considerably responsive to increase by alteration of the dairy cow diet (typically to 30–32 g/100 g total fatty acids), and this is accompanied by a reduction in SFA typically from 70 g/100 g total fatty acids to 50–55 g/100 g total fatty acids (Kliem et al., 2019). Moreover, there is now increasing evidence that replacement of SFA by MUFA in the human diets provides beneficial blood lipid profiles and other benefits (Sellem et al., 2022; Weech et al., 2018), thus providing a rationale for partial replacement of SFA with MUFA in dairy foods. This topic is covered in detail by Markey and Kliem (2020) and highlights that whilst many studies have examined changing milk fat composition by modifying the diet of dairy cows, few human RCTs have been done to assess whether these modifications provide health benefits.

Nevertheless, an RCT of this type was recently undertaken at the University of Reading in the ‘REplacement of SaturatEd fat in dairy on Total cholesterol’ (*RESET*) study (ClinicalTrials.gov NCT02089035), a project funded by the UK Medical Research Council. This study investigated the effect of replacing a proportion of SFA in milk and subsequent cheese and butter on blood lipids, aspects of vascular function and a wide range of CVD risk biomarkers, after both acute and chronic consumption. Kliem et al. (2019) reported results on the fatty acid composition of the dairy foods used and the outcome of the 12‐week chronic intervention study by Vasilopoulou et al. (2020). Table 5 summarises some key outcomes from this human study which show that consuming the modified dairy foods led to significant attenuation of fasting LDL‐C rise; a lower LDL‐C:HDL‐C ratio, improved vascular endothelial function (increased %FMD) and increased plasma nitrite concentration. There was no effect on HDL and LDL sub‐classes, arterial stiffness or BP. This study was therefore not restricted to changes in blood lipids, and the technology may have potential as a public health strategy to reduce CVD risk. Of course, it is not known if the benefits arose equally from all three modified dairy foods involved, although from other studies discussed earlier, it is likely that changes to the fatty acids in the butter would have greater effect than in the cheese.

**TABLE 5:** Some key outcomes from the 12‐week chronic RCT in the RESET study comparing dairy foods with a proportion of SFA replaced with monounsaturated fatty acids (Modified) with conventional dairy foods (Control) from Vasilopoulou et al. (2020)

	Modified dairy foods	Control dairy foods	*p*
	∆ from baseline	∆ from baseline	
Fasting serum cholesterol			
TC (mmol/L)	0.12 ± 0.07	0.29 ± 0.06	0.08
LDL‐C (mmol/L)	0.03 ± 0.06	0.19 ± 0.05	0.03
HDL‐C (mmol/L)	0.04 ± 0.02	0.07 ± 0.02	0.55
LDL‐C:HDL‐C ratio	−0.06 ± 0.04	0.05 ± 0.04	0.04
TC:HDL‐C ratio	−0.03 ± 0.05	−0.04 ± 0.03	0.13
Endothelial function			
% FMD	0.35 ± 0.06	−0.51 ± 0.15	<0.0001
Pre‐occlusion artery diameter (mm)	0.06 ± 0.04	0.04 ± 0.04	0.08
Peak artery diameter (mm)	0.07 ± 0.04	−0.07 ± 0.04	0.02
Time to peak diameter (s)	−0.5 ± 1.9	4.5 ± 2.4	0.04
CBEAI			
Nitrite (μmol/L)	0.02 ± 0.01	−0.03 ± 0.02	0.01
VCAM‐1 (ng/ml)	3.0 ± 20.7	40.1 ± 1.7	0.08

*Note*: Values are unadjusted means ± SEM.

Abbreviations: CBEAI, circulating biomarkers of endothelial activation and inflammation; FMD, flow‐mediated dilatation; HDL‐C, high‐density lipoprotein cholesterol; LDL‐C, low‐density lipoprotein cholesterol; RCT, randomised controlled trial; TC, total cholesterol; VCAM‐1, vascular cell adhesion protein 1; ∆, change.

## OVERALL CONCLUSIONS

Many of the prospective studies and RCTs included in this short review do not support a simple positive association between SFA intake and the risk of atherosclerotic CVDs and its components. Those studies that included blood cholesterol data do broadly support the positive relationship between SFA and LDL‐C but without a consistent subsequent relationship with increased CVD risk, despite LDL being a causal factor in atherosclerotic CVDs. This suggests that LDL‐C alone is not a consistently good risk predictor or cause of CVDs, and this is highly relevant to the health effects of dairy foods. Some reasons for this include:

1. The potentially counterbalancing effect of any concomitant increase in HDL‐C.
2. Food matrix factors, which reduce fat absorption and/or alter the cholesterol efflux capacity of HDL as noted for some dairy foods.
3. SFA intake reduction not leading to a reduction in the more atherogenic small, dense LDL particles, although there remains some doubt about the consistency of this effect.
4. Considerable variation in serum LDL‐C response to SFA between individuals.
5. The body's exposure to SFA (16:0 in particular) being substantially influenced by in vivo synthesis, predominantly from carbohydrates, especially sugars.
6. Counterbalancing food‐related health effects on improved vascular function (e.g. lower BP).

Not all of the above processes are fully understood or validated in an adequate number of studies which makes the translation of these into public health nutrition policy difficult, although several groups have provided convincing arguments that dietary recommendations should be food‐based and, for example, not limited to overall restrictions on SFA intake (e.g. Astrup et al., 2020; Krauss & Kris‐Etherton, 2020). It is clear that dairy foods are not associated with increased CVD risk, despite being key sources of SFA, perhaps because of some combinations of the above factors, and it is noteworthy that some non‐dairy studies have also shown LDL‐C reduction not being reflected in reduced CVD risk. Overall, restrictions on dairy food intake do not seem warranted on health grounds, although there are suggestions that supply may be reduced on sustainability grounds. There remains a need to further understand the association between dairy food types and chronic diseases, perhaps particularly for type 2 diabetes. It seems that saturated fats, dairy foods and health are increasingly less of a curious paradox.

## CONFLICT OF INTEREST

The author has been a consultant to the Estonian Bio‐competence Centre of Healthy Dairy Products (BioCC) and to the UK Dairy Council on fats in dairy products and cardiometabolic disease and in addition has received travel expenses and honorariums in connection with meetings and lectures from the Dairy Council, Dutch Dairy Association, European Dairy Association and the International Dairy Federation.

## Data Availability

There are no data specifically from this review paper but all accepted publications are loaded to the University of Reading's depository Centaur which is publicly accessible at https://centaur.reading.ac.uk/.
